# Editorial: The Impact of Music on Human Development and Well-Being

**DOI:** 10.3389/fpsyg.2020.01246

**Published:** 2020-06-17

**Authors:** Graham F. Welch, Michele Biasutti, Jennifer MacRitchie, Gary E. McPherson, Evangelos Himonides

**Affiliations:** ^1^Department of Culture, Communication and Media, University College London, London, United Kingdom; ^2^Department of Philosophy, Sociology, Education and Applied Psychology, University of Padua, Padua, Italy; ^3^School of Humanities and Communication Arts, Western Sydney University, Penrith, NSW, Australia; ^4^Melbourne Conservatorium of Music, University of Melbourne, Melbourne, VIC, Australia

**Keywords:** music, wider benefits, lifespan, health, well-being

Music is one of the most universal ways of expression and communication for humankind and is present in the everyday lives of people of all ages and from all cultures around the world (Mehr et al., 2019). Hence, it seems more appropriate to talk about musics (plural) rather than in the singular (Goble, 2015). Furthermore, research by anthropologists as well as ethnomusicologists suggests that music has been a characteristic of the human condition for millennia (cf. Blacking, 1976; Brown, 1999; Mithen, 2005; Dissanayake, 2012; Higham et al., 2012; Cross, 2016). Nevertheless, whilst the potential for musical behavior is a characteristic of all human beings, its realization is shaped by the environment and the experiences of individuals, often within groups (North and Hargreaves, 2008; Welch and McPherson, 2018). Listening to music, singing, playing (informally, formally), creating (exploring, composing, improvising), whether individually and collectively, are common activities for the vast majority of people. Music represents an enjoyable activity in and of itself, but its influence goes beyond simple amusement.

These activities not only allow the expression of personal inner states and feelings, but also can bring about many positive effects in those who engage in them. There is an increasing body of empirical and experimental studies concerning the wider benefits of musical activity, and research in the sciences associated with music suggests that there are many dimensions of human life—including physical, social, educational, psychological (cognitive and emotional)—which can be affected positively by successful engagement in music (Biasutti and Concina, 2013). Learning in and through music is something that can happen *formally* (such as part of structured lessons in school), as well as in *other-than-formal* situations, such as in the home with family and friends, often non-sequentially and not necessarily intentional, and where participation in music learning is voluntary, rather than mandated, such as in a community setting (cf. Green, 2002; Folkestad, 2006; Saether, 2016; Welch and McPherson, 2018).

Such benefits are evidenced across the lifespan, including early childhood (Gerry et al., 2012; Williams et al., 2015; Linnavalli et al., 2018), adolescence (McFerran et al., 2018), and older adulthood (Lindblad and de Boise, 2020). Within these lifespan perspectives, research into music's contribution to health and well-being provides evidence of physical and psychological impacts (MacDonald et al., 2013; Fancourt and Finn, 2019; van den Elzen et al., 2019). Benefits are also reported in terms of young people's educational outcomes (Guhn et al., 2019), and successful musical activity can enhance an individual's sense of social inclusion (Welch et al., 2014) and social cohesion (Elvers et al., 2017).

This special issue provides a collection of 21, new research articles that deepen and develop our understanding of the ways and means that music can impact positively on human development and well-being. The collection draws on the work of 88 researchers from 17 different countries across the world, with each article offering an illustration of how music can relate to other important aspects of human functioning. In addition, the articles collectively illustrate a wide range of contemporary research approaches. These provide evidence of how different research aims concerning the wider benefits of music require sensitive and appropriate methodologies.

In terms of childhood and adolescence, for example, Putkinen et al. demonstrate how musical training is likely to foster enhanced sound encoding in 9 to 15-year-olds and thus be related to reading skills. A separate Finnish study by Saarikallio et al. provides evidence of how musical listening influences adolescents' perceived sense of agency and emotional well-being, whilst demonstrating how this impact is particularly nuanced by context and individuality. Aspects of mental health are the focus for an Australian study by Stewart et al. of young people with tendencies to depression. The article explores how, despite existing literature on the positive use of music for mood regulation, music listening can be double-edged and could actually sustain or intensify a negative mood.

A Portuguese study by Martins et al. shifts the center of attention from mental to physical benefits in their study of how learning music can support children's coordination. They provide empirical data on how a sustained, 24-week programme of Orff-based music education, which included the playing of simple tuned percussion instruments, significantly enhanced the manual dexterity and bimanual coordination in participant 8-year-olds compared to their active control (sports) and passive control peers. A related study by Loui et al. in the USA offers insights into the neurological impact of sustained musical instrument practice. Eight-year-old children who play one or more musical instruments for at least 0.5 h per week had higher scores on verbal ability and intellectual ability, and these correlated with greater measurable connections between particular regions of the brain related to both auditory-motor and bi-hemispheric connectivity.

Younger, pre-school children can also benefit from musical activities, with associations being reported between informal musical experiences in the home and specific aspects of language development. A UK-led study by Politimou et al. found that rhythm perception and production were the best predictors of young children's phonological awareness, whilst melody perception was the best predictor of grammar acquisition, a novel association not previously observed in developmental research. In another pre-school study, Barrett et al. explored the beliefs and values held by Australian early childhood and care practitioners concerning the value of music in young children's learning. Despite having limited formal qualifications and experience of personal music learning, practitioners tended overall to have positive attitudes to music, although this was biased toward music as a recreational and fun activity, with limited support for the notion of how music might be used to support wider aspects of children's learning and development.

Engaging in music to support a positive sense of personal agency is an integral feature of several articles in the collection. In addition to the Saarikallio team's research mentioned above, Moors et al. provide a novel example of how engaging in collective beatboxing can be life-enhancing for throat cancer patients in the UK who have undergone laryngectomy, both in terms of supporting their voice rehabilitation and alaryngeal phonation, as well as patients' sense of social inclusion and emotional well-being.

One potential reason for these positive findings is examined in an Australian study by Krause et al.. They apply the lens of self-determination theory to examine musical participation and well-being in a large group of 17 to 85-year-olds. Respondents to an online questionnaire signaled the importance of active music making in their lives in meeting three basic psychological needs embracing a sense of competency, relatedness and autonomy.

The use of public performance in music therapy is the subject of a US study by Vaudreuil et al. concerning the social transformation and reintegration of US military service members. Two example case studies are reported of service members who received music therapy as part of their treatment for post-traumatic stress disorder, traumatic brain injury, and other psychological health concerns. The participants wrote, learned, and refined songs over multiple music therapy sessions and created song introductions to share with audiences. Subsequent interviews provide positive evidence of the beneficial psychological effects of this programme of audience-focused musical activity.

Relatedly, McFerran et al. in Australia examined the ways in which music and trauma have been reported in selected music therapy literature from the past 10 years. The team's critical interpretive synthesis of 36 related articles led them to identify four different ways in which music has been used beneficially to support those who have experienced trauma. These approaches embrace the use of music for stabilizing (the modulation of physiological processes) and entrainment (the synchronization of music and movement), as well as for expressive and performative purposes—the fostering of emotional and social well-being.

The therapeutic potential of music is also explored in a detailed case study by Fachner et al.. Their research focuses on the nature of critical moments in a guided imagery and music session between a music therapist and a client, and evidences how these moments relate to underlying neurological function in the mechanics of music therapy.

At the other end of the age span, and also related to therapy, an Australian study by Brancatisano et al. reports on a new *Music, Mind, and Movement* programme for people in their eighties with mild to moderate dementia. Participants involved in the programme tended to show an improvement in aspects of cognition, particularly verbal fluency and attention. Similarly, Wilson and MacDonald report on a 10-week group music programme for young Scottish adults with learning difficulties. The research data suggest that participants enjoyed the programme and tended to sustain participation, with benefits evidenced in increased social engagement, interaction and communication.

The role of technology in facilitating access to music and supporting a sense of agency in older people is the focus for a major literature review by Creech, now based in Canada. Although this is a relatively under-researched field, the available evidence suggests that that older people, even those with complex needs, are capable of engaging with and using technology in a variety of ways that support their musical perception, learning and participation and wider quality of life.

Related to the particular needs of the young, children's general behavior can also improve through music, as exampled in an innovative, school-based, intensive 3-month orchestral programme in Italy with 8 to 10-year-olds. Fasano et al. report that the programme was particularly beneficial in reducing hyperactivity, inattention and impulsivity, whilst enhancing inhibitory control. These benefits are in line with research findings concerning successful music education with specific cases of young people with ADHD whose behavior is characterized by these same disruptive symptoms (hyperactivity, inattention, and impulsivity).

Extra-musical benefits are also reported in a study of college students (Bachelors and Masters) and amateur musicians in a joint Swiss-UK study. Antonini Philippe et al. suggest that, whilst music making can offer some health protective effects, there is a need for greater health awareness and promotion among advanced music students. Compared to the amateur musicians, the college music students evaluated their overall quality of life and general and physical health more negatively, as did females in terms of their psychological health. Somewhat paradoxically, the college students who had taken part in judged performances reported higher psychological health ratings. This may have been because this sub-group were slightly older and more experienced musicians.

Music appears to be a common accompaniment to exercise, whether in the gym, park or street. Nikol et al. in South East Asia explore the potential physical benefits of synchronous exercise to music, especially in hot and humid conditions. Their randomized cross-over study (2019) reports that “time-to-exhaustion” under the synchronous music condition was 2/3 longer compared to the no-music condition for the same participants. In addition, perceived exertion was significantly lower, by an average of 22% during the synchronous condition.

Comparisons between music and sport are often evidenced in the body of existing Frontiers research literature related to performance and group behaviors. Our new collection contains a contribution to this literature in a study by Habe et al.. The authors investigated elite musicians and top athletes in Slovenia in terms of their perceptions of flow in performance and satisfaction with life. The questionnaire data analyses suggest that the experience of flow appears to influence satisfaction with life in these high-functioning individuals, albeit with some variations related to discipline, participant sex and whether considering team or individual performance.

A more formal link between music and movement is the focus of an exploratory case study by Cirelli and Trehub. They investigated a 19-month-old infant's dance-like, motorically-complex responses to familiar and unfamiliar songs, presented at different speeds. Movements were faster for the more familiar items at their original tempo. The child had been observed previously as moving to music at the age of 6 months.

Finally, a novel UK-based study by Waddington-Jones et al. evaluated the impact of two professional composers who were tasked, individually, to lead a 4-month programme of group composing in two separate and diverse community settings—one with a choral group and the other in a residential home, both funded as part of a music programme for the Hull City of Culture in 2017. In addition to the two composers, the participants were older adults, with the residential group being joined by schoolchildren from a local Primary school to collaborate in a final performance. Qualitative data analyses provide evidence of multi-dimensional psychological benefits arising from the successful, group-focused music-making activities.

In summary, these studies demonstrate that engaging in musical activity can have a positive impact on health and well-being in a variety of ways and in a diverse range of contexts across the lifespan. Musical activities, whether focused on listening, being creative or re-creative, individual or collective, are infused with the potential to be therapeutic, developmental, enriching, and educational, with the caveat provided that such musical experiences are perceived to be engaging, meaningful and successful by those who participate.

Collectively, these studies also celebrate the multiplicity of ways in which music can be experienced. Reading across the articles might raise a question as to whether or not any particular type of musical experience is seen to be more beneficial compared with another. The answer, at least in part, is that the empirical evidence suggests that musical engagement comes in myriad forms along a continuum of more or less overt activity, embracing learning, performing, composing and improvising, as well as listening and appreciating. Furthermore, given the multidimensional neurological processing of musical experience, it seems reasonable to hypothesize that it is perhaps the level of emotional engagement in the activity that drives its degree of health and well-being efficacy as much as the activity's overt musical features. And therein are opportunities for further research!

## Author Contributions

The editorial was drafted by GW and approved by the topic Co-editors. All authors listed have made a substantial, direct and intellectual contribution to the Edited Collection, and have approved this editorial for publication.

## Conflict of Interest

The authors declare that the research was conducted in the absence of any commercial or financial relationships that could be construed as a potential conflict of interest.
